# Seclusion in an enriched environment versus seclusion as usual: A quasi-experimental study using mixed methods

**DOI:** 10.1371/journal.pone.0259620

**Published:** 2021-11-11

**Authors:** Cornelia G. J. M. van der Venne, Berno van Meijel, Mathijs Deen, Miranda Olff, Cornelis L. Mulder

**Affiliations:** 1 Parnassia Psychiatric Institute, The Hague, The Netherlands; 2 Inholland University of Applied Sciences, Amsterdam, The Netherlands; 3 Department of Psychiatry, Amsterdam UMC, Amsterdam Public Health Research Institute, Amsterdam, The Netherlands; 4 Department of Psychiatry, Amsterdam UMC, Amsterdam Neuroscience and Amsterdam Public Health, Amsterdam, The Netherlands; 5 ARQ National Psychotrauma Centre Diemen, Diemen, The Netherlands; 6 Antes Parnassia Psychiatric Institute, Rotterdam, The Netherlands; Queensland University of Technology, AUSTRALIA

## Abstract

**Background:**

For patients, seclusion during psychiatric treatment is often a traumatic experience. To prevent such experiences, adjustments in the design of seclusion rooms have been recommended.

**Methods:**

As there have been no empirical studies on the matter, we used a quasi-experimental design to compare the experiences in seclusion of two groups of patients: 26 who had been secluded in a room designed according to the principles of healing environment, a so called ‘Enriched Environment Seclusion room’ (EES), and 27 who had been secluded in a regular seclusion (RS) room. The enrichment included audio-visual facilities, a fixed toilet, a couch and a self-service system to adjust light, colour, blinds and temperature according to the patient’s preferences. Insight into their experiences was obtained using the Patient View-of-Seclusion Questionnaire, which comprises nine statements on seclusion, supplemented with open-ended questions.

**Results:**

The responses regarding seclusion experiences between the two groups did not differ significantly (*U* = 280.00, *p* = .21, *r* = -.17). Although those who had been secluded in the specially designed room had greatly appreciated the opportunities for distraction, and those who had been secluded in a regular seclusion room expressed the need for more distracting activities during seclusion, both groups described seclusion as a dreadful experience. If seclusion cannot be avoided, patients recommend facilities for distraction (such as those provided in an enriched environment seclusion room) to be available.

**Conclusion:**

Whatever the physical environment and facilities of a seclusion room, we may thus conclude that seclusion is a burdensome experience.

## Introduction

Until recently, eclusion often was considered to be an inevitable intervention for very severely agitated psychiatric inpatients. It was justified on the basis of three premises: containment, to ensure safety for the patient and/or others,isolation and the reduction of sensory input to mitigate illness-induced responses to environmental stimuli [1,2].

At the time that Sailas and Fenton (2009) [3] published a review in which no controlled trials were identified that provided evidence for the therapeutic effectiveness of seclusion, a growing awareness of the negative effects of seclusion emerged.It became clear that seclusion is likely to have a range of negative effects, such as anger, powerlessness, punishment, loneliness, harmfulness, fear of confined spaces, feelings of helplessness, and the risk of traumatisation or re-traumatisation [4–10]. In addition, witnessing other patients being secluded is likely to induce feelings of distress [11,12].

Although awareness of the negative impact of seclusion has led to wide-ranging research on patients’ experiences of seclusion [6,7,13–16] to the development of new interventions to prevent seclusion [17–20], there is also sceptism. Stakeholders like patients, professionals, researchers and policymakers argue that seclusion cannot be completely eliminated, and should thus be available as a measure of last resort [8,12,21–23].

As we are worldwide in a period of transition–possibly towards the total elimination of seclusion–but total abolition has not yet been imposed, it is in patients’ interest to prevent the traumatic experiences they can undergo during seclusion. For this reason, considerable attention has been paid in recent years to implementing interventions on staff-patient interactions [24–26]. Improvements to the environmental design of inpatient wards in general with interdisciplinary input such as architecture and environmental psychology [27–29] have been evaluated and, more specifically, the design of wards with a seclusion [30]. Recent studies have also examined the effect on patients of psychiatric intensive care units (PICU) [31], and those of customized environments where psychiatric patients can stay in cases of crisis or imminent crisis, such as ‘comfort rooms’ with a particular focus on preventing seclusion [16,32]. In a controlled trial, Lloyd et al (2014) [15] showed that seclusion rates declined significantly on wards that offered the use of comfort rooms to patients with signs of increasing distress. Similarly, in a study that described 14 design characteristics of closed wards that significantly affected the risk of being secluded, van der Schaaf et al (2013) [33] showed that this risk was reduced by the availability of ‘total private space per patient’, a higher ‘level of comfort’ and ‘greater visibility’ (good overview) on the ward’. They concluded that these three aspects were associated with privacy and autonomy of patients and had positive effects on patients’ well-being.

Various studies have recommended environmental modifications to the design of seclusion rooms, such as comfortable furniture, access to music, radio, television, adequate toilet facilities and the ability to control the room’s temperature [12]; options for meaningful activities such as reading magazines or books [9]; and a large window that makes it possible to look outside and benefit from natural light [34]. However, we have found no studies on how a differently designed room may affect patients’ experiences of seclusion. In this quasi-experimental study we therefore wished to compare the experiences of two groups of patients: those who had been secluded in a so-called ‘healing environment’ seclusion room, and those who had been secluded in a regular seclusion room. Healing environment is understood as the embedding of environmental variables with a positive influence on recovery and well-being and comprises the location of the building, the view, the building itself, the furnishing and the offered facilities [27,29,35]. We hypothesized that the feelings experienced by patients secluded in a healing-environment seclusion room would be less negative than those experienced by patientsin shaping secluded in a regular seclusion room.

## Methods

### Design

We used a quasi-experimental study design (see procedures) to compare the experiences of two groups of patients: those who had been secluded in a seclusion room designed according to the principles of a healing environment (Enriched Environment Seclusion, EES), and those who had been secluded in a regular seclusion room (RS).

### Setting and participants

**Fig 1:**
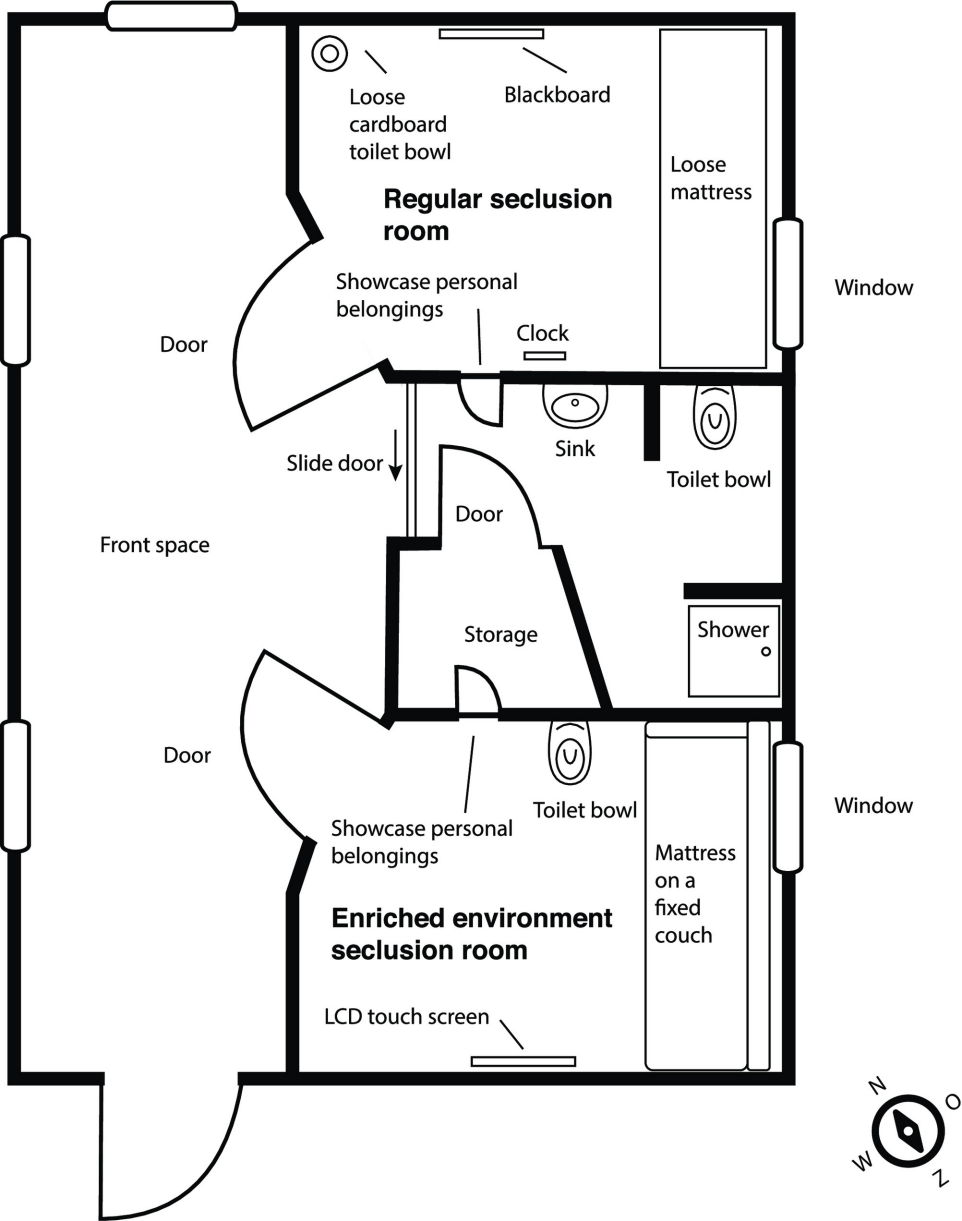
Floor plan and facilities seclusion area.

The study was conducted in a large mental health Institution in the western part of the Netherlands, in three closed forensic psychiatric wards where judicial imposed treatments are given for crisis and regular admissions. The wards were equiped with one EES and RS as shown in Fig 1.

The design of the EES was based on the awareness of the psychological impact of the environment, summarized as healing environment by a Dutch environmental psychologist de Vos [29] and described in the evaluation instrument “Oazis-tool” developed by van der Schaaf [36]. The tool comprises the following themes: privacy and autonomy, windows and view, comfort and controle, facilities and utilities, orientation and routing, interior, nature, staff. The new design also included the recommendations of patients who had been secluded in the past. This is in line with the recommandations by Chrysikou (2015) [37] and de Vos (2006) [29] to always design buildings and environments in consultation with the end users. As a result, the EES had the following facilities, which, except for the window, were not available in the regular seclusion room: (1) a touchscreen that allowed the patient to control the room temperature, the blinds, and the colour and intensity of the lighting as well as playing games and playing videos (for example a forest with bird sounds or a beach with the sounds of waves and seagulls); (2) more comfortable furniture, comprising a mattress on a couch, a wood pattern on the floor, and a metal toilet bowl with flushing mechanism. In contrast, the RS was a bare room with a matress and a window, paper containers rather than a water closet, and a blackboard for writing or drawing. Neither did the RS provide the patient with options for adjusting the environment to his or her personal needs. Table 1 lists the facitilies of the two seclusion rooms.

**Table 1:** Summary of seclusion room facilities.

Facilities	EES*	RS**
Adjustable music, video themes, games	+	-
Adjustable temperature, light and blinds	+	-
Comfortable couch	+	-
Water closet	+	-
Writing and drawing	+	+
Window	+	+
Visible personal belongings	+	+

*EES = Enriched Environment Seclusion room

**RS = Regular Seclusion room.

### Patient view-of-seclusion questionnaire

The Patient View-of-Seclusion Questionnaire was used to gain insight into the patients’ experiences during the seclusion period because it fulfilled two important conditions: the most common feelings during seclusion are included and the needed time for completion by the subjects is limited. This brief questionnaire [6], consists of five negatively formulated statements and four positively formulated statements regarding seclusion.The responses to the items are scored on a 5-point Likert rating scale, ranging from 1 = ‘not at all’ to 5 = ‘very much’. After its translation to Dutch, the questionnaire had been backtranslated by a sworn Dutch-English translator, and then discussed and finalised by the researchers. It had a Cronbach’s alpha of 0.86, indicating good internal consistency.

### Open-ended questions

To obtain more detailed insight into patients’ personal experiences during the seclusion period, open-ended questions were added to the Patient View-of-Seclusion Questionnaire. These focused on positive and negative aspects and experiences of the seclusion episode and on the facilities in the seclusion room. See the appendix for these questions.

### Collection of demographic and clinical data

Data on demographics and diagnoses were collected from the patients’ electronic clinical data files. The patients original DSM-IV TR diagnoses had been made by the ward psychiatrists on the basis of a clinical interview.

### Procedures

The determining factor for allocating patients to the EES or RS was the availability of the EES; if a patient had to be secluded and the EES was already occupied by another patient, the new patient was secluded in the RS, irrespective of his or her patient characteristics. Given this random allocation, no patient-related selection procedures were applied to either the EES or the RS, thereby ensuring the comparability of the two patient groups. This approach implied that seclusion in the EES or the RS was independent from participation in the study-interview. After a patient released from seclusion, department staff would inform the researcher. To avoid inclusion bias, all patients who had the capacity to consent (as indicated by the psychiatrist on the ward on the basis of a psychiatric evaluation) were recruited for research participation which meant that patients who were not approachable due to their pscyhiatic state were excluded. Patients were then asked to participate in the study. Once written informed consent had been given, the Patient View-of-Seclusion Questionnaire was administered by an independent interviewer in a private area at the ward. The interviewer was not involved in the research but could not be blinded for the seclusion condition. No staff member was present during the interview.We The response categories (5-point Likert scale) were shown to the patients on a printed page. To obtain more detailed insight into the patients’ experiences after completion of the questionnaire, the same interviewer conducted a short semi-structured interview based on the open-ended questions. In order to avoid contamination of the data, only one seclusion episode per patient was considered for data collection. We did nog exclude patients with seclusion experiences prior to admission. The average admission time of one year at the 16-beds ward thus determined the limited inclusion of patients al the start of the study. Later in the study two similar wards with both seclusion conditions became available for our study, which accelerated the inclusion of patients significantly. At the same time, institutional efforts to reduce seclusion in general slowed down the data collection process.

### Power assumptions

The power analysis was based on our hypothesis that the patients’ responses to the Patient View-of-Seclusion Questionnaire would show that the EES had had a positive effect on their experience of the seclusion period. Given that the EES had been equipped on the basis not only of studies that had recommended similar improvements to seclusion rooms, but also of inputs from patients who had previously been secluded, we expected the responses to the Patient View-of-Seclusion Questionnaire to produce a higher score for the EES than for the RS. With an alpha level of .05, a power of .80 and a high effect size (Cohen’s *d* = .8) [38], the required number of participants per condition was 26.

### Data analysis

To test our primary hypothesis after transforming the scores of the negatively formulated statements, we analysed the quantitative data by comparing the total sum scores of the Patient View-of-Seclusion Questionnaire. To compare the effects of the EES and the RS more in depth we then conducted exploratory analyses using the single items of the questionnaire. Next, to compare the experimental and control group for sum scores and single-item scores, we performed a non-parametric Mann-Whitney U-test (the total sum scores of the Patient View-of Seclusion Questionnaire were not normally distributed). An alpha of 5% was used for all tests. After verbatim transcription of the qualitative interview data, we conducted thematic analysis in order to obtain greater insight into patients’ experiences during seclusion. This qualitative research method describes experiences, meanings and the reality in patients’ own words [39].

### Ethics

The research protocol was approved by the Medical Ethical Committee for Research in Mental Health Care in the Netherlands (METIGG) and was conducted in accordance with the Declaration of Helsinki. Patients were included after written informed consent had been obtained.

## Results

### Background characteristics

Of 104 patients who met the inclusion criteria between January 2011 and September 2016, 53 patients participated in the study and were distributed equally over the EES and RS conditions. Reasons for non inclusion were not willing to participate (N = 16), language difficulties (N = 3), disease related (N = 3), voluntarily secluded (N = 1), patient from another division (N = 1), not recruited after seclusion (N = 5) and discharged when researcher was informed about eligibility (N = 22). The average period of time between the end of the seclusion episode and the interview was 15 days. This time duration was biased by the fact that the researcher was informed by the staff. As a result, the patients could not be asked for the assessment immediately after the determination of seclusion. Table 2 shows the patient characteristics for the two conditions. There were no significant differences between the two groups. The high number of men in both groups was consistent with the sex distribution of the patients admitted to the closed wards in question.

**Table 2:** Patient characteristics.

	N	EES (n = 26)	RS (n = 27)
Age, mean (sd)	53	34 (9)	39 (11)
Male gender, n (%)	53	21 (81)	24 (89)
Diagnoses (N = 53)			
Psychotic disorders, n (%)	30	17 (65)	13 (48)
Substance-related disorder, n (%)	10	4 (15)	6 (22)
Personality disorder, n (%)	5	3 (12)	2 (7)
Pervasive developmental disorder, n (%)	3	1 (4)	2 (7)
Mood disorder, n (%)	3	1 (4)	2 (7)
Other/deferred, n (%)	2	0	2 (7)
Reason for seclusion (N = 47)			
Aggression/disruptive behaviour, n (%)	42	20 (87)	22 (92)
Suicide risk n (%)	3	2 (9)	1 (4)
Provide rest n (%)	2	1 (4)*	1 (4)
Seclusion period in hours, mean (sd)	53	88 (110)	72 (152)
Days interviewed after seclusion, mean (sd)	53	13 (15)	17 (19)
Seclusion experience before admission, n (%)	50	23 (46)	24 (48)

*Music calmed patient down.

### Patient view-of-seclusion questionnaire

A Mann-Whithney-U-test showed no significant differences between the two conditions with regard either to the sum scores of the Patient View-of-Seclusion Questionnaire: EES (*Mdn* = 28) *and* RS (*Mdn* = 23) (*U* = 280.00, *p =* .21, *r* = -.17), or to the separate items. Table 3 compares the two groups at item level.

**Table 3:** Separate answers per item.

Patient View-of-Seclusion statements	Median EES (n = 26)	Median RS (n = 27)	*U*	*p*	*r*
1 This seclusion caused me harm	2	4	310.0	.454	-.10
2 This seclusion calmed me	4	4	301.5	.358	-.13
3 This seclusion made me anxious	2	4	286.0	.233	-.16
4 This seclusion protected me	3	3	351.0	1.0	0
5 This seclusion made me angry	3	4	309.0	.439	-.11
6 This seclusion helped me	4	2	315.5	.511	-.09
7 I experienced this seclusion as a punishment	4	4	328.5	.671	-.06
8 This seclusion made me feel safe	3	2	311.5	.470	-.10
9 This seclusion made me sad	3	4	253.0	.071	-.25

### Open-ended questions

The qualitative analysis was conducted by extracting the issues which the patients referred to most after the seclusion episode. Their experiences proved to be clustered around three themes: ‘feelings about the stay in the seclusion room’, ‘the seclusion episode as a whole’, and ‘distraction during the seclusion episode’.

#### Theme 1: Feelings about the stay in the seclusion room

Negative feelings with respect to seclusion were described by a vast majority in both groups (74% and 76% had negative feelings with respect to the RS and EES, respectively). Although only patients who had stayed in the RS complained about the uncomfortable cardboard toilet and the cold room temperature, patients in both groups stated that negative feelings had been caused by being separated from others and by the lack of contact with other people. In other words, isolation had had a decisive impact on their experience of seclusion. A patient who had stayed in the RS stated ‘I didn’t like it. I don’t want to be alone; when you’re alone, you no longer exist.’ Patients who had been secluded in the EES explained that, despite the new facilities, they still felt locked up and isolated. They described it as follows: ‘it’s still seclusion’; ‘even though there’s a computer, you’re still locked up: you can’t leave, and you feel you’re isolated from other people; ‘the fact that the door was closed and nobody was there made me delusional’; and ‘the loneliness caused voices in my head.’ Patients in both groups gave similarly general descriptions of the feelings caused by the seclusion episode, three of them being ‘frightening,’ ‘terrible’ and ‘heavy.’ Stronger expressions for their dismay were ‘I’d have preferred corporal punishment’ (RS) and ‘it was the worst thing that has ever happened in my life’ (EES).

#### Theme 2: The seclusion episode as a whole

With regard to the seclusion episode as a whole (i.e. from the moment it was decided that coercion was necessary), many patients had felt humiliated or punished by the staff’s treatment, and also by the mandatory seclusion wear, the lack of privacy, and the large number of people involved in the episode. Patients who had been secluded in the RS expressed it as follows: ‘The fact that you had to lie on the floor, that you had to wear certain clothes–my feeling was that it was just to belittle people’; and ‘it’s so humiliating the way you’re taken to the seclusion room, with six people grabbing you, and being stripped naked’; or ‘animals get better treatment.’ One of the subjects who had been secluded in the EES explained that ‘the treatment was very patronizing, like I was a small child.’ Another patient who had been secluded in the EES said, ‘I didn’t like the way the staff spoke to me, and I had no privacy when undressing.’ Whatever the design of the seclusion room, it was clear that these experiences were stressful.

#### Theme 3: Distraction during a seclusion episode

Patients who had been secluded in the RS indicated that distraction would be a major improvement, as it would help alleviate the loneliness and help the time pass more quickly. Television, books, magazines, newspapers, games, and music were often mentioned in this context; patients who had been secluded in the RS said ‘a screen to watch television on [would make you] feel less alone and help the time pass faster’ and ‘a radio to listen to the news to know what was going on in the world [would make] the time pass quicker’. Patients who had stayed in the EES mostly appreciated the facilities that offered distraction during their stay in the seclusion room; they stated that the EES was a significant improvement on the RS. Many explained that the music had had a calming and soothing effect. A patient who had stayed in the EES said: ‘I felt less locked up because I was distracted from the problems that had come up.’ The woodland theme with singing birds helped another patient to calm down because ‘it reminded me of my parakeet at home.’ Most patients suggested that the EES facilities be extended, for example with more variety in music styles, more challenging games, and books or audio books.

## Discussion

This study used a quasi-experimental design to investigate whether the experience of seclusion had been less negative for patients who had been secluded in a room designed according to the principles of a healing environment (EES) than it had been for patients secluded in a regular seclusion (RS) room. Unexpectedly, the quantitative data derived from the Patient View-of-Seclusion Questionnaire showed no statistically significant differences between the seclusion-room experiences of the two groups, either at sum level or at item level. Patients from both groups described the seclusion episode as unpleasant and negative. However, the distraction provided in the healing-environment seclusion room had helped to make the episode more bearable.

To our knowledge, this is the first study to examine the impact of a redesigned seclusion room on seclusion experiences. Our results confirm earlier findings that seclusion is a terrifying and invasive intervention [4,6,11,12]. Patients’ overall experience of seclusion is determined partly by factors such as hospital staff’s interpersonal conduct towards patients; staff’s unequal position of power (since they dictate what the patient has to do); infringements of human dignity (with regard to the lack of privacy); and the physical discomfort caused by uncomfortable seclusion wear which is made of stiff material. In short, it is, as Hoekstra et al (2004) [7] described, an impressive event, where the difference positions of patients and staff put the patients’ autonomy is at stake.

As our study shows, even if the facilities in a seclusion room are consistent with the principals of a healing environment and designed in consultation with the endusers a seclusion episode is perceived as burdensome. It might therefore be concluded that the stress caused by seclusion is caused largely by factors independent of the conditions in the seclusion room. The results of the qualitative data suggest that the highest negative impact was caused by the experience of being locked up. Both groups of patients indicated that being isolated from others and losing contact with others gave rise to negative feelings about the seclusion episode. This fact is important because the end users contributed to the design precisely in order to avoid a mismatch between the design and the end users, as Chrysikou (2015) [37] recommends. Although more in-depth research should take place, it is a significant indication of the severity of a seclusion episode and the negative impact of social deprivation. The isolation in itself may have equal or perhaps even more significant effect on the impact of the experience than physical environmental factors. To put this finding into a broader perspective, it could be argued that this is consistent with the description made by Juan Mendez on behalf of the United Nations in 2011 when equating confinement with torture: ‘Considering the severe mental pain or suffering solitary confinement may cause, it can amount to torture or cruel, inhuman or degrading treatment… .” [40].

As a new intervention in the EES, we added audio-visual stimuli as one of the aspects of a healing environment. In the interviews that followed their seclusion, patients who had been secluded in an RS recommended distraction as an improvement, while those who had had access to such stimuli in the EES had greatly appreciated them. These statements seem to confirm the assumptions expressed in earlier research that distraction could mitigate seclusion experiences [8,9,32]. This implies that distraction should be offered to patients who still need to be secluded in the transition period toward the total elimination of seclusion worldwide. Subsequently, in-depth research into the efficacy of distraction should be done. This can lead to further improvement of the design of seclusion rooms, to be used in inevitable cases, and to more options to prevent seclusion. Distraction could have such a calming effect that seclusion is no longer necessary. The results can therefore contribute to their further integration in the use of comfort rooms [15], high-intensity care units [31] and the division of wards as described by van der Schaaf [33].

## Limitations

Our results should be viewed in perspective. First, since the sample size was relatively small, and the patients were recruited in only one hospital, care is necessary when generalising the results. In other words, our findings can be confirmed only in future research that has a larger sample size and is conducted in a range of hospital settings. However, statistically and more important clinically significant differences are not guarantied with a larger sample. Second, as the majority of patients in our sample were male, our study also gives little insight into the seclusion experiences of women. Third, as our results stem from a forensic population, caution is also needed when generalizing these results to the general psychiatric population. Keski Valkama et al (2010) [8] found that forensic patients experienced seclusion as a punishment significantly more often than patients of a general psychiatric group. This could have impacted the total sum scores of the View-of-Seclusion Questionnaire. A fourth limitation concerns the actual moment of the interview which was approximately two weeks after the seclusion period. Importantly however, in this regard there was no significant difference between both seclusion conditions. A final limitation may be the use of the Patient View-of-Seclusion Questionnaire as the most important outcome measure. During the development of this instrument, optimal face validity was pursued by Hammill et al (1989) [6] through patient experiences of seclusion found in the literatur. However, no further psychometric tests have been conducted.For future research it could be valuable if the questionnaire would be tested more thoroughly on its psychometric properties, in order to further develop the quality of the instrument. In addition, this questionnaire does not have specific items on the use and benefits of distraction, which in the qualitative part of this study turned out to be important.

## Conclusions

Our study showed that the experiences of patients who had been secluded in a room designed according to the principles of a healing environment were not less negative than those of patients secluded in a regular seclusion room. While the patients in the EES valued the facilities provided to distract them from the experience of seclusion, these facilities did not provide an overall experience of seclusion that was significantly better. Our qualitative data showed the importance of distraction during a crisis situation that resulted in a seclusion episode. To help individual patients to release from a crisis situation without having to be secluded, future research to the essence and functioning of distraction is recommended.

In sum, our study indicates that seclusion is a burdensome experience regardless of the design or the offered facilities in a seclusion room. It supports the internationally felt need to diminish seclusion and when needed, the enriched environment seclusion room may make it more bearable. In order to be prepared for a future without seclusion, more preventive measures such as the use of comfort rooms [32] or High Intensive Care Units [16] need to be implemented.

## Supporting information

S1 File Open-ended questions. (DOCX)

## Abbreviations

EES: a seclusion room based on the principles of healing environment
RS: regular seclusion room
METIGG: Medisch-ethische Toetsingscommissie Instellingen Geestelijke Gezondheidszorg
