# Supplementary material for: Seclusion in an enriched environment versus seclusion as usual: A quasi-experimental study using mixed methods
Source: PLoS One. 2021 Nov 11;16(11):e0259620. doi: 10.1371/journal.pone.0259620 (PMC8584674; doi:10.1371/journal.pone.0259620)
Supplement: S1 File — (DOCX) [file pone.0259620.s001.docx]

**Supporting information**

**Open-ended questions**

1. What of the total seclusion episode impressed you most?
2. How did you experience this?
3. How did you experience the entire seclusion period?
4. What did you like about the seclusion period and what didn’t you like?
5. In your view, which aspects of the seclusion period should be changed?
6. What did you like about the seclusion room and what didn’t you like?
7. In your view, how should the seclusion room be changed?
8. In one sentence, how would you summarize your experience of seclusion?
